# Evaluation of the Expression of Matrix Metalloproteinase-1 of Laryngeal Squamous Cell Carcinoma by Ultrasound Molecular Imaging

**DOI:** 10.3389/fphar.2019.00655

**Published:** 2019-06-19

**Authors:** Yi Zhou, Zhuqing Song, Qiao Hu, Xiaojuan Ji, Hongyu Zheng, Xiaoyan Wang, Zhenzhou Li

**Affiliations:** ^1^GuangZhou University School of Medicine, GuangZhou, China; ^2^Department of Breast Surgery, Peking University Shenzhen Hospital, Shenzhen, China; ^3^Department of Ultrasound, The People’s Hospital of Guangxi Zhuang Autonomous Region, Nanning, China; ^4^Department of Cardiology, Children’s Hospital of Chongqing Medical University, Chongqing, China; ^5^Department of Ultrasound, The Second People’s Hospital of Shenzhen, The First Affiliated Hospital of Shenzhen University, Shenzhen, China

**Keywords:** ultrasound molecular imaging, matrix metalloproteinase-1, laryngeal squamous cell carcinoma, targeted microbubbles, vasculogenic mimicry

## Abstract

**Purpose:** The aims of this study were to evaluate the expression of matrix metalloproteinase-1 (MMP-1) on laryngeal squamous cell carcinoma (LSCC) and improve the early diagnosis rate *via* ultrasound molecular imaging (USMI).

**Methods:** The microsized MMP-1-targeted microbubbles (MB_MMP-1_) and the control MBs (MB_IgG_) based on perfluorocarbon-filled lipid-shelled MBs were constructed and characterized. The *in vitro* binding experiment was performed with human epidermoid laryngeal cancer cells (HEp-2) and tested the binding efficiency of MB_MMP-1_ and MB_IgG_. In the *in vivo* study, the LSCC model was established in 10 mice. The MB_MMP-1_ and MB_IgG_ were randomly injected into tumor-bearing mice *via* the tail vein at Day 7, Day 12, and Day 17 to dynamically evaluate the differential targeted enhancement (dTE) signals *via* USMI. Subsequent immunofluorescence analysis was used for confirmation of MMP-1 expression.

**Result:** The effective adhesion rate of MB_MMP-1_ and MB_IgG_ to HEp-2 was 298.42 ± 16.57 versus 12.38 ± 3.26 bubbles/per field *in vitro* experiment, which shows a significant difference (*P* < 0.01). The *in vivo* ultrasound molecular imaging (USMI) results demonstrated that dTE signal intensity from MB_MMP-1_ was significantly higher than that from the MB_IgG_ at Day 7, Day 12, and Day 17 (Day 7, 41.21 ± 15.00 versus 2.25 ± 0.6 a.u., *P* < 0.05; Day 12, 124.64 ± 5.19 versus 11.13 ± 1.13 a.u., *P* < 0. 05; Day 17, 332.01 ± 64.88 versus 42.99 ± 11.9 a.u., *P* < 0.01). Moreover, immunofluorescence analysis further confirmed the expression of MMP-1 in LSCC with a gradual increase with the tumor growth.

**Conclusion:** MB_MMP-1_ could be a potential probe that can be used in the early diagnosis of LSCC by USMI.

## Introduction

Laryngeal squamous cell carcinoma (LSCC) is one of the common malignant tumors of the head and neck. Recent data from global epidemiology of head and neck cancers show that more than 245,000 new cases of laryngeal cancer will be expected by 2030 (Gupta et al., 2016). Although great significant progress has made in the diagnosis, its early diagnosis rate of LSCC still remains to be raised. Microbubble-based ultrasound molecular imaging (USMI) shows significant potential in the early diagnosis of tumors. Ultrasound contrast agents (UCAs) such as microbubbles (MBs) can emit significantly stronger acoustic signals under an appropriate sonic energy excitation, making them several thousand times more reflective than normal body tissues. Through designing ultrasonic contrast agents with specific molecular markers, USMI probes can be obtained and used to visualize molecular and genetic alterations of diseased cells, and to monitor the genesis and development of certain diseases. Previously, researchers have demonstrated that the MBs combined with antibodies or peptides, which can bind to vascular endothelial growth factor (VEGR) or αvβ3 integrin, and enhanced the contrast ultrasonography quality in LSCC *via* USMI (Paolo et al., 2006; Hu et al., 2016). However, in malignant LSCC tumors, the expression of EGFR or αvβ3 integrin was commonly not at a high level (Kumar, 2003; Gino et al., 2010).

Matrix metalloproteinase-1 (MMP-1), a member of the family of MMPs, plays crucial roles in vascular formation and remodeling *via* degrading vascular membrana basilaris and extracellular matrix (ECM) proteins (Raffetto and Khalil, 2008). In addition, several studies have confirmed that the MMP-1 is specially associated with LSCC growth, local invasion, and metastasis (Tsukifuji et al., 1999). Upile et al., (2011) had claimed that the angiogenesis of LSCC was not only a unique means to nourish tumor tissues. Specially, it generated “micro-vascular” channels without the composition of endothelial cells at the early stage. This event was known as vascular mimicry (VM) in which endothelial-like cells are transdifferentiated by their own stem cells. More importantly, both angiogenesis and VM as a part of tumor microenvironment are coordinately providing tumor initiation and progression, and the latter one even seriously contributed to the invasion of LSCC (Upile et al., 2011; Balkwill et al., 2012). In view of above-mentioned reasons, we believed that the biomarker of MMP-1 would provide a potential evaluation of its progress in LSCC biology, thus allowing for the design of new diagnostics and therapeutics for early cancer diagnosis and treatment.

To date, the positive rate of conventional medical imaging examination, such as computed tomography (CT) and magnetic resonance imaging (MRI), in detecting LSCC is still low in detection of LSCC (Castelijns and Mw, 1993; Yamazaki et al., 2008; Norling et al., 2014; Righi et al., 2015). USMI is a multifunctional and famous medical imaging tool for the detection and visualization of cancer-related biomarkers in early disease diagnosis (Pysz et al., 2010; Yan et al., 2011; Feng et al., 2013; Wood and Sehgal, 2015). In the past decades, MBs with different types of shells composed of phospholipids or polymers and gas cores (perfluorocarbon, nitrogen, sulfur hexafluoride, or air) had been applied in USMI and performed good contrast enhancement (Kiessling et al., 2009; Abou-Elkacem et al., 2016). Some special biomarkers of ligands such as integrin and Vascular cell adhesion molecule-1 (VCAM-1) or EGFR can be tightly bound on the surface of MBs for a better specificity diagnosis (Fabian et al., 2012). Recently, it has been successfully applied to various diseases such as vascular plaque and tissue inflammation, but its potential use in the diagnosis of LSCC has not been reported. In our study, we preliminarily developed the MB_MMP-1_ and dynamically evaluated the expression of MMP-1 separately at Day 7, Day 12, and Day 17 of LSCC progression *in vivo* by USMI.

## Materials and Methods

### Preparation of MB_MMP-1_

MB_MMP-1_ and control MB_IgG_ were prepared according to a previous report (Stieger et al., 2010). In brief, DSPC: DSPE-PEG2000:DSPE-PEG2000-biotin (Avanti Polar Lipids, Alabaster, AL, USA) (molar ratios = 9:0.5:0.5) was blended in chloroform, and the solvent was removed under nitrogen flow at room temperature, followed by vacuum treatment over 2 h. The dried blends were hydrated at 60°C with phosphate-buffered saline (PBS) and sub-packaged into vials (1 mL each vial). After that, perfluoropropane (C_3_F_8_; Flura, Newport, TN, USA) was added, and the admixture was mechanically vibrated for 45 s. After that, MB_MMP-1_ contrast group was prepared by incubating these biotinylated MBs with excess avidin, followed by adding a given proportion (50 μg × 10^8^ MBs) of biotinylated anti-mouse MMP-1 monoclonal antibody (ဂNovus Biologicals, Colorado, USA). Incubation at room temperature for 15–30 min and washing three to four times by centrifugation (400*g*) were necessary for avidin or antibody linkage. The biotinylated IgG antibody (Novus Biologicals, Colorado, USA) was used instead of MMP-1 to connect with biotinylated MBs in the same way to obtain MB_IgG_ in the control group.

### Characterization of MB_MMP-1_

The surface morphology of MB_MMP-1_ was investigated using a microscope (Leica DMI3000 B). Fluorescent microscopic examination was performed to verify the anti-mouse MMP-1 monoclonal primary antibody conjugation efficiency of MB_MMP-1_ according to fluorescent intensity of fluorescein isothiocyanate (FITC)-conjugated goat anti-mouse second antibody (St. Louis, MO, USA) under a fluorescent microscope (Olympus, Tokyo, Japan). MB_MMP-1_ average size, distribution, and differential intensity were evaluated three times for each sample using a diameter limit of 0.5 μm (AccuSizer 780; Particle Sizing Systems, Santa Barbara, CA, USA).

### Cell Culture

Human epidermoid laryngeal cancer cells (Procell Life, Wuhan, China) (HEp-2) were cultured in Dulbecco’s modified Eagle medium (DMEM) supplemented with 10% fetal bovine serum (FBS), 1% l-glutamine, and 1% penicillin–streptomycin. The cell cultures were maintained in a humidified atmosphere of 5% CO_2_ at 37°C with the medium changed every other day.

### Tumor Model

All animal studies were approved by the Institute’s Animal Care and Use Committee of Shenzhen Second People’s Hospital, Guangzhou University School of Medicine, China. The methods were carried out in accordance with the approved guidelines. Nude male Balb/c mice (6- to 8-week-old, body weight 20 ± 1.5 g) were supplied by Beijing Vital River Laboratory Animal Technology Co. Ltd. All mice were housed on a 12:12 light:dark cycle with free access to food and water. HEp-2 cells (1 × 10^6^/each mouse) dissolved in 100 μL of PBS were injected into the left oxter of nude male Balb/c mice. Tumor-bearing mice were used for USMI when they had developed at Day 7, Day 12, and Day 17 after inoculation.

### *In Vitro* Imaging Performance of MB_MMP-1_

MB_MMP-1_ with concentrations of 1 × 10^5^, 1 × 10^6^, and 1 × 10^7^ MBs/mL was placed in agar gel holes. The US imaging performance of MBs was detected from the side of the agar gel with VisualSonics Vevo2100 (VisualSonics, Inc., Toronto, Canada) to determine the intensity of the ultrasound signal of the MBs with concentrations of 1 × 10^5^, 1 × 10^6^, and 1 × 10^7^ MBs/mL.

### Binding Specificity of MB_MMP-1_ to Human Epidermoid Laryngeal Cancer Cells

In a 6-well plate (1 × 10^5^ cells per well), 1 × 10^5^ HEp-2 cells were cultured overnight. The next day, the culture medium was discarded, and the cells were washed three times with PBS, and then 1 × 10^8^ MBs/mL of MB_MMP-1_ or MB_IgG_ was added to the cells and incubated to the plate for 5–6 min. After being washed three to five times with PBS, the binding efficiency of MB_MMP-1_ and control MB_IgG_ to cells was examined under an inverted microscope (Olympus, Tokyo, Japan). At the same time, the competitive binding inhibition experimental group (pre-blocking MMP-1 receptors by adding an excess of free MMP-1 monoclonal antibody) was also set up to study the binding specificity of MB_MMP-1_ to HEp-2.

### *In Vivo* Ultrasound Molecular Imaging

USMI was conducted as described previously (Stieger et al., 2010). Briefly, the mice were kept anesthetized by continuous inhalation of 2% isoflurane in oxygen at 2 L/min on a heated stage during scanning. US imaging was performed using a dedicated small-animal high-resolution USMI system (Vevo2100, VisualSonics) equipped with an 18-MHz high-frequency nonlinear transducer. All imaging parameters (grain, 30 dB; focal depth, 2–4 mm; transmit power, 10%; MI, 0.1) were kept constant during all imaging sessions. MB_MMP-1_ or MB_IgG_ (5 × 10^8^ MBs in 200 μL of PBS) was administered *via* the tail vein of the mice in random order to minimize bias, and injections were separated by at least 30 min to allow clearance of MBs from the blood circulation. To distinguish the acoustic signal from MBs that had adhered to MMP-1 receptors from the signal from freely circulating MBs, a destruction/replenishment approach was used in this study. In brief, after injection of MBs and waiting for 7 min, approximately 200 ultrasonographic frames of the tumor were acquired at a temporal resolution of 12 s. Then a high-power ultrasound destruction sequence was applied for 1 s to destroy the MBs. After the destruction pulse, another set of movie (≈200 frames) was acquired. Movie processing and quantification were performed using Vevo2100 built-in software of CQ and relying on two sets of movie: a pre-destruction set and a post-destruction (background) data set. The post-destruction movie signals were subtracted from the pre-destruction signals. The difference in movie signal intensity between pre-destruction and post-destruction ultrasonographic frames was calculated and expressed as movie intensity amplitude.

### Imaging Data Analysis

The imaging data sets of all mice were analyzed by Vevo2100 built-in software CQ. Regions of interest were drawn covering the entire area of the tumor. The movie signal intensity from attached MBs was assessed by calculating an average for pre-destruction and post-destruction imaging signals and subtracting the average post-destruction signal from the average pre-destruction signal. The subtracted signal, so-called differential targeted enhancement (dTE), was colored red and then displayed as a colored overlay on the contrast-mode images.

### Immunofluorescence

Tumor-bearing mice were euthanized after USMI, and the subcutaneous tumors were excised, embedded in optimal cutting temperature compound, and frozen in dry ice. Frozen blocks were sectioned at 5 μm (CM1950, Leica, Heidelberg, Germany) and mounted on glass slides for immunofluorescence staining. A double-staining procedure was employed to visualize MMP-1 expression on tumor cells. The following method was used for mouse MMP-1 staining. First, paraffin sections were successively dewaxed with xylene I for 15 min and xylene II for 15 min; hydrated with absolute ethanol I for 5 min, absolute ethanol II for 5 min, 85% ethanol for 5 min, and 75% ethanol for 5 min; and afterwards washed with distilled water. Second, these were subsequently blocked with 5% goat serum for 30 min at room temperature. And then the slides were co-incubated with rabbit anti-mouse MMP-1 primary antibody with dilution 1:1,500 ratio (Google Biotechnology Co., Ltd, Wuhan, China) overnight at 4°C and visualized by using Cy3-conjugated goat anti-rabbit second antibody (Servicebio, Wuhan, China) in a 1:300 ratio (Google Biotechnology Co., Ltd, Wuhan, China). Next, the slides were placed in PBS (pH 7.4) on as decolorization shaking table for 5 min. After the drying process, dihydrochloride (DAPI) dye was added to the slides, and samples were protected from light and incubated for 10 min. Finally, fluorescent images were acquired at ×400 magnifications with a laser scanning confocal microscope (TCS SP5, Leica, Germany). And then the correlation analysis between the signal intensity of USMI and mean optical density (/pixel) of MMP-1 expression was performed separately at Day 7, Day 12, and Day 17.

### Statistical Analysis

Quantitative data were expressed as the means and standard deviation (mean ± SD). Data from two independent samples were analyzed with Student’s *t* test. Analysis of variance (ANOVA) was used to determine the significance of differences in multiple comparisons. A *P* value of less than 0.05 was considered statistically significant. Statistical analyses were performed using Statistical Product and Service Solutions (SPSS) software version 13.0 (SPSS Inc, Chicago, IL, USA).

## Results

### Characterization of MB_MMP-1_

**Figure 1:**
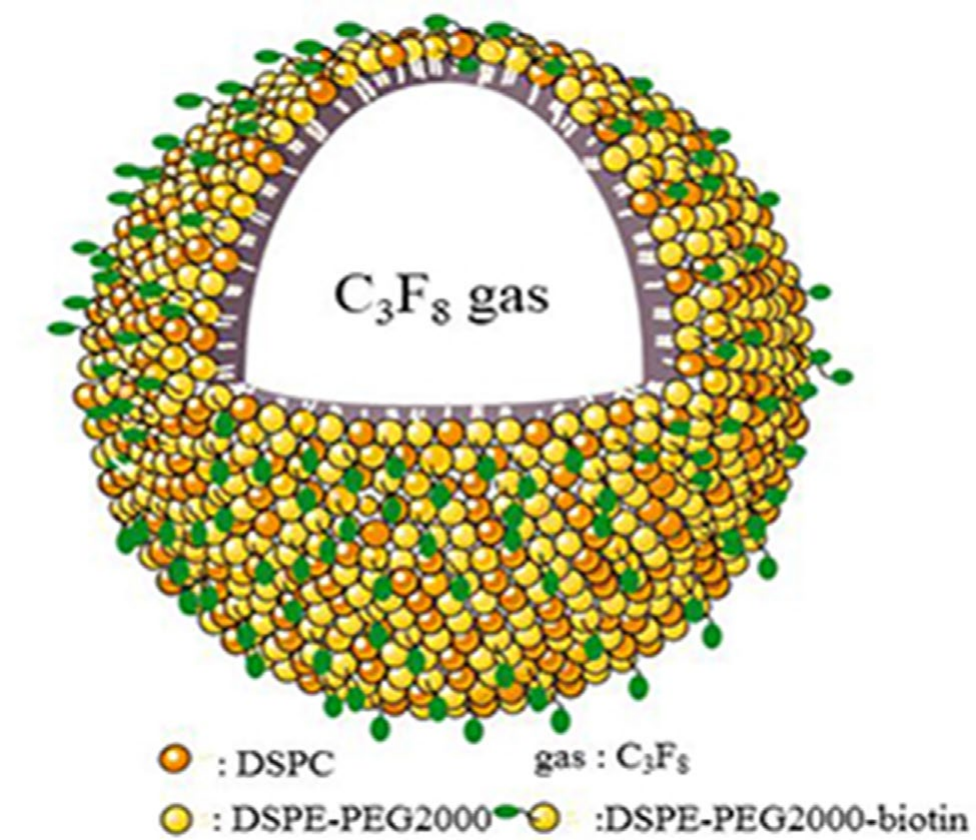
Schematic illustration of MB_MMP-1_. DSPC = distearoylphosphatidylcholine; DSPE = distearoylphosphatidylethanolamine; PEG2000 = polyethylene glycol 2000.

**Figure 2:**
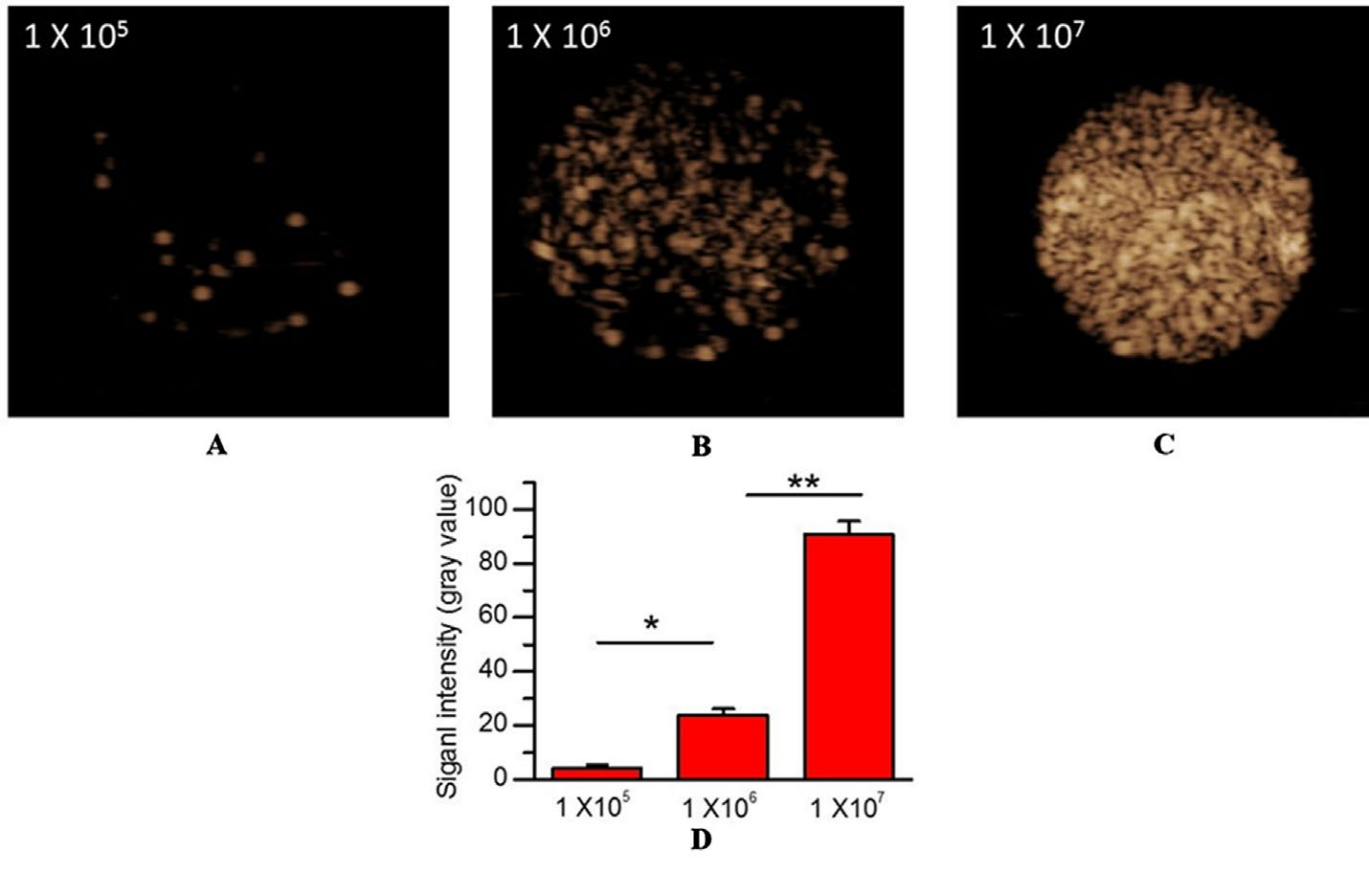
In vitro US imaging performance. **(A)** 1×105 MBs/mL. **(B)** 1×106 MBs/mL. **(C)** 1×107 MBs/mL. **(D)** Quantitative analysis of US signal intensity of MBMMP-1 with three different concentrations.

**Figure 1** shows a schematic illustration of MB_MMP-1_. Microscopic image analysis at high magnification revealed that the resulting MB_MMP-1_ has regular spherical morphology, transparent center, and good dispersion with no adherence to each other (**Figure 2A**). The FITC-conjugated MB_MMP-1_ shows bright green fluorescence under fluorescence microscope (**Figure 2B**), indicating the successful conjugation of anti-MMP-1 antibodies onto the surface of MBs. Mean size distributions of the MB_MMP-1_ are shown in **Figure 2C** by AccuSizer 780, revealing that the mean diameter of the MB_MMP-1_ was centered at 1.11 ± 0.10 µm.

### *In Vitro* Imaging Performance of MB_MMP-1_

**Figure 3:**
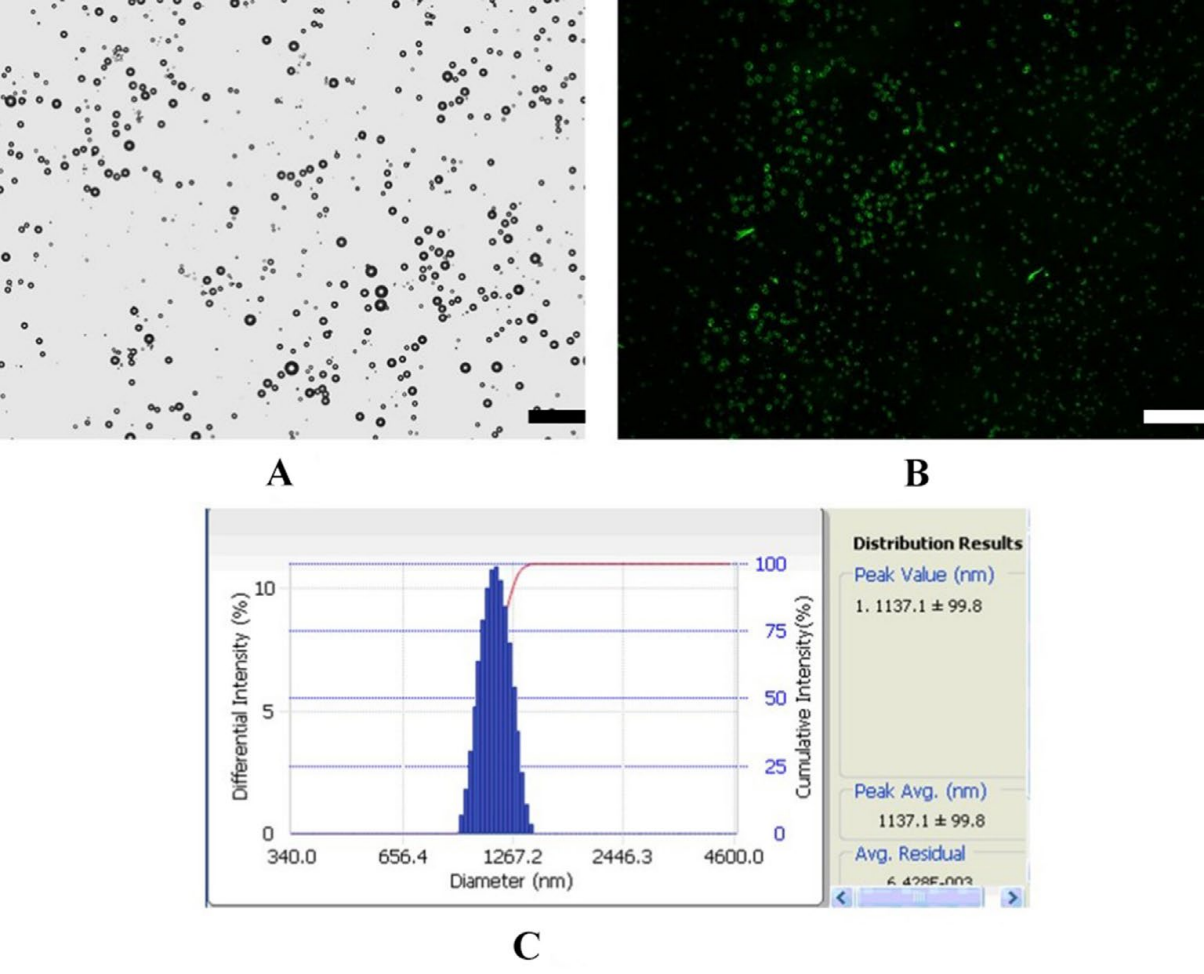
Characterization of MB_MMP-1_. **(A)** Bright-filed photograph of MB_MMP-1_. **(B)** Fluorescent micrograph of FITC-labeled MB_MMP-1_. **(C)** Average size and distribution of the MB_MMP-1_ (scale bar = 10 µm).

To confirm the imaging performance of MB_MMP-1_ as a contrast agent for USMI, three different concentrations of MB_MMP-1_ were assessed *in vitro via* contrast imaging mode (**Figure 3A, B, C**). We found the three samples’ echoes are relatively well distributed. In addition, with the increase of the concentration of MB_MMP-1_, the signal intensity of contrast imaging increases. In particular, we found that the best performance of contrast imaging was at 1 × 10^7^ MBs/mL. Then, we quantified the average signal intensity of different concentrations of MB_MMP-1_, revealing 4.11 ± 1.37, 24.03 ± 2.06, and 90.72 ± 4.92 a.u. at 1 × 10^5^, 1 × 10^6^, and 1 × 10^7^ MBs/mL, respectively (**Figure 3D**).

### Binding Specificity of MB_MMP-1_ to Human Epidermoid Laryngeal Cancer Cells

**Figure 4:**
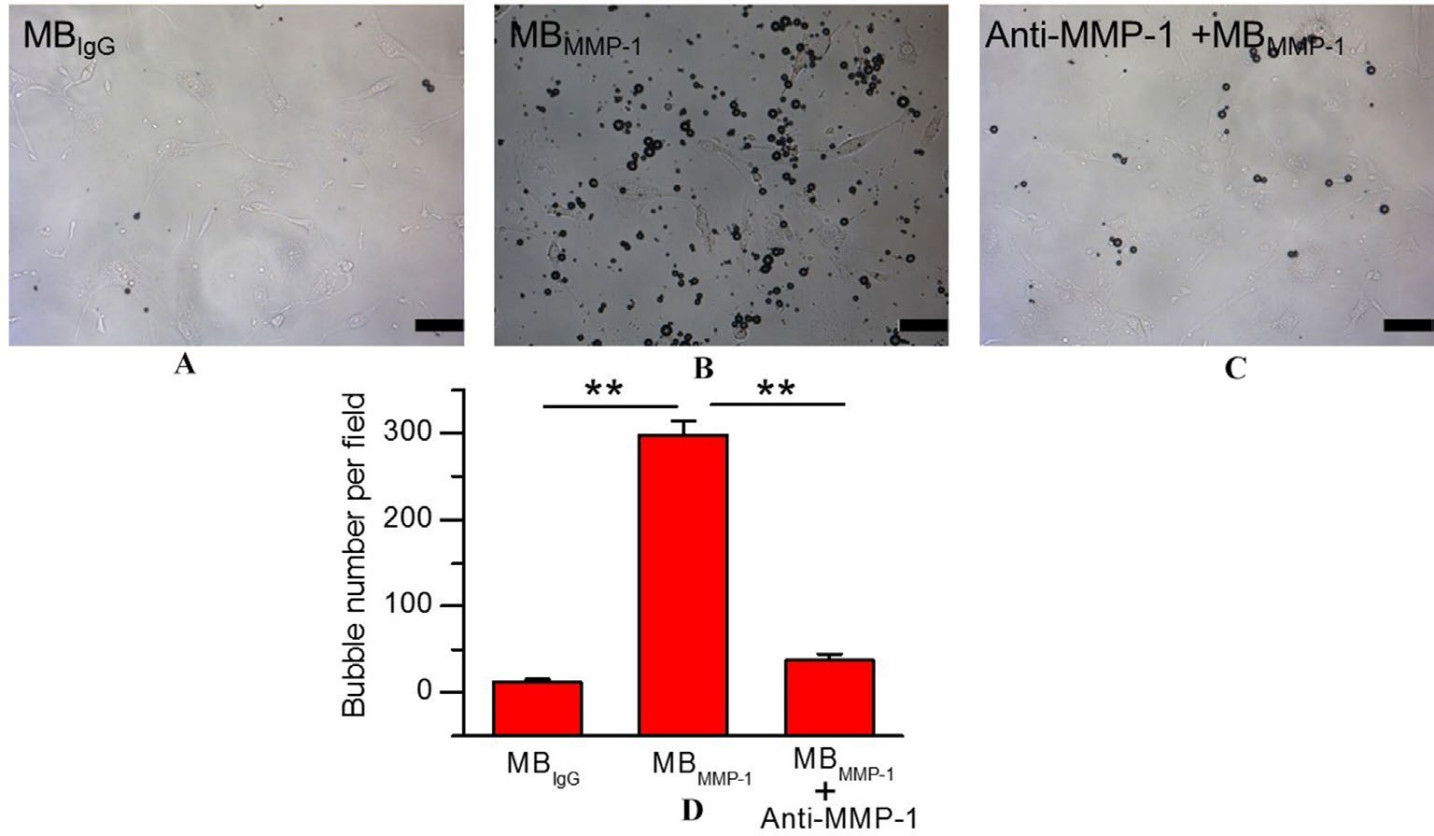
*In vitro* binding specificity experiment of MB_MMP-1_ to human epidermoid laryngeal cancer cells (HEp-2). **(A)** White light micrograph after incubating control group MB_IgG_ with HEp-2 cells. **(B)** White light micrograph after incubating MB_MMP-1_ with HEp-2 cells. **(C)** White light micrograph after incubating pre-blocked with free anti- matrix metalloproteinase-1 (MMP-1) antibody. **(D)** Quantitative analysis of the number of MB_MMP-1_ and control group MB_IgG_ that adhered onto HEp-2 from five random view fields (**P < 0.01, *n* = 5). Scale bar = 10 µm.

To ensure the binding specificity of the MB_MMP-1_, we further conducted the binding specificity of MB_MMP-1_ or MB_IgG_ with the HEp-2 by comparing with the group that was pre-incubated with free anti-MMP-1 antibodies for 5–6 min. In bright field, our results (**Figure 4A**) showed that the control MB_IgG_ only had a small number of non-targeted binding specificity on the HEp-2 surface with 12.38 ± 3.26 MBs/per field, while the MB_MMP-1_ could specifically bind to the HEp-2 with 298.42 ± 16.57 MBs/per field (**Figure 4B**) (298.42 ± 16.57 versus 12.38 ± 3.26 MBs/per field, *P* < 0.01). And as for the pre-blocking group by an excess of free anti-MMP-1 monoclonal antibody, it was found that the number of MB_MMP-1_ attached to HEp-2 was significantly decreased with 37.3 ± 6.94 MBs/per field (**Figure 4C**) (298.42 ± 16.57 versus 37.3 ± 6.94 MBs/per field, *P* < 0.01). Quantitative analysis indicated that the binding specificity of MB_MMP-1_ was significantly about 20 times higher than that of control MB_IgG_ (298.42 ± 16.57 versus 12.38 ± 3.26, **P < 0.01) (**Figure 4D**).

### USMI *In Vivo*

**Figure 5:**
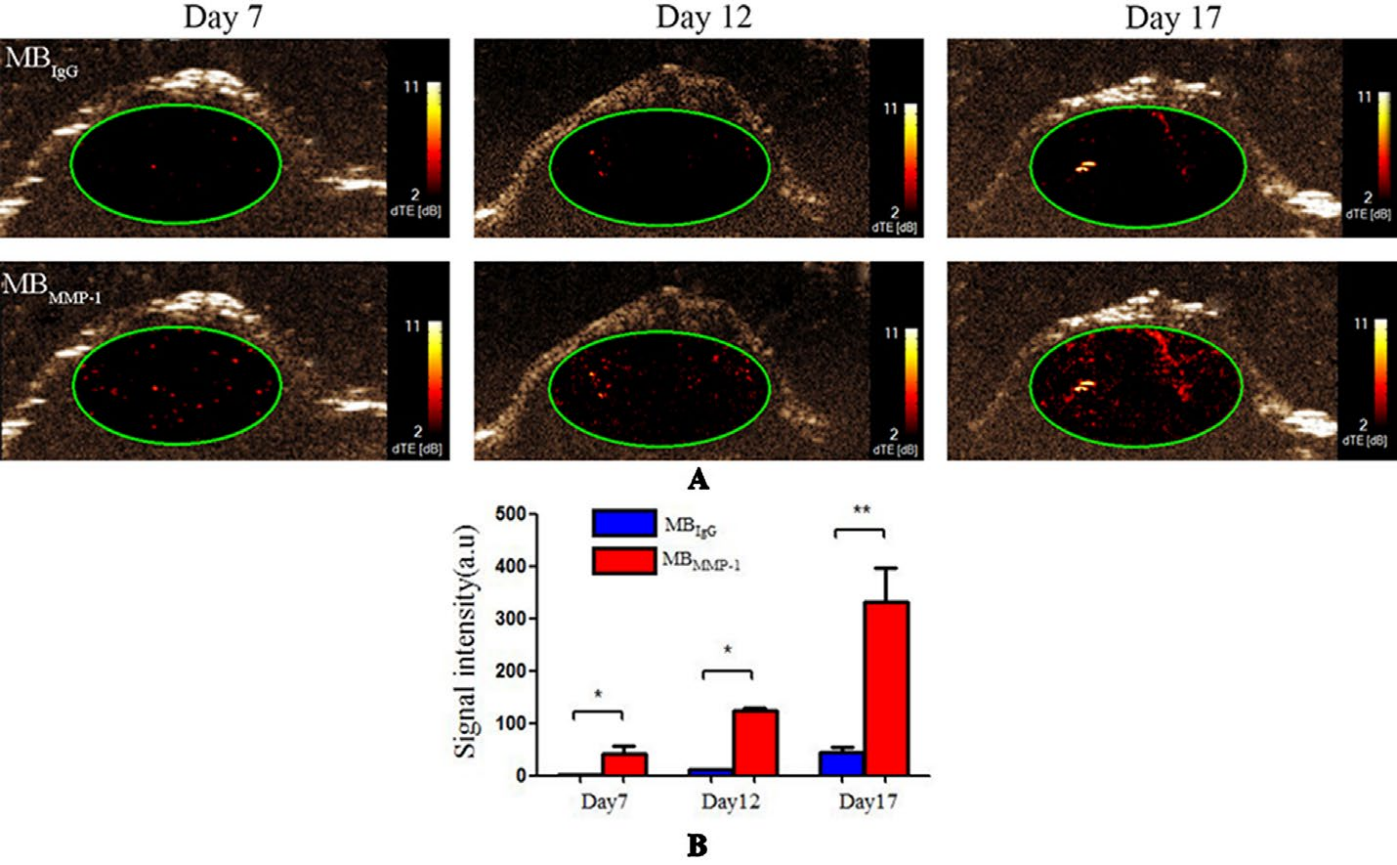
Ultrasound molecular imaging (USMI) *in vivo*. **(A)** Color-coded differential targeted enhancement (dTE) signal sonograms separately at Day 7, Day 12, and Day 17. **(B)** Quantitative analysis of dTE for the MB_MMP-1_ and control group MB_IgG_ (*P < 0.05, **P < 0.01).

MB_MMP-1_ and MB_IgG_ were further evaluated in tumor-bearing mice at Day 7, Day 12, and Day 17 by USMI. The injections of MB_MMP-1_ or MB_IgG_ were separated by at least 30 min. From **Figure 5A**, we could clearly observe that the MB_MMP-1_ of color red had more retention rate in tumors and significantly enhanced the US imaging signal intensity, while the control MB_IgG_ showed a significantly lower enhancement than did MB_MMP-1_. In addition, with the growth of LSCC, the dTE signal intensity was much higher in MB_MMP-1_. From **Figure 5B**, we plotted the US dTE signal intensity from the LSCC versus growth time of the Day 7, Day 12, and Day 17. There were 20-, 10- and 8-folds higher dTE signal intensity from MB_MMP-1_ than control MB_IgG_. Then we quantify the dTE signal intensity with Vevo2100 inner software CQ. Quantification revealed 41.21 ± 15.00 a.u. for MB_MMP-1_ versus 2.25 ± 0. 6 a.u. for MB_IgG_ at Day 7 (*P < 0.05), 124.64 ± 5.19 a.u. MB_MMP-1_ versus 11.13 ± 1.13 a.u. MB_IgG_ at Day 12 (*P < 0.05), and 332.01 ± 64.88 a.u. MB_MMP-1_ versus 42.99 ± 11. 9 a.u. MB_IgG_ at Day 17 (**P < 0.01).

### Tumor Immunofluorescence Staining

**Figure 6:**
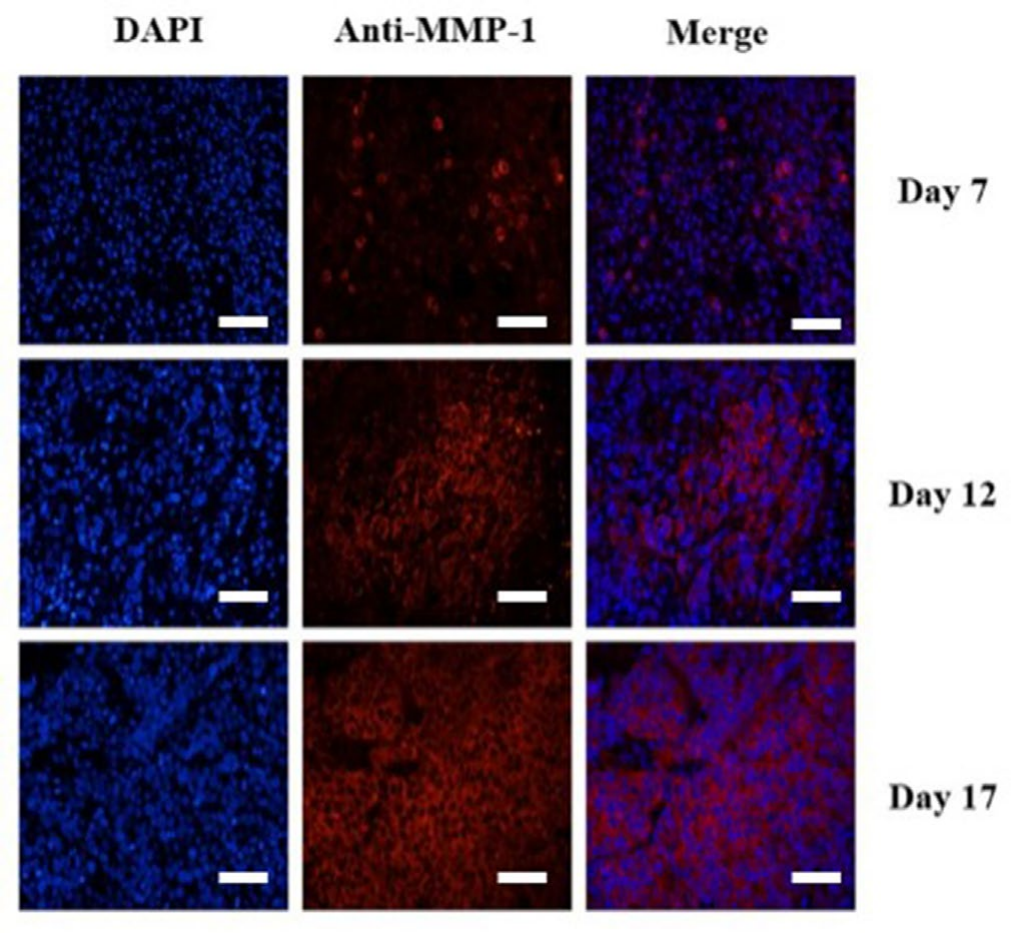
Immunofluorescence staining of HEp-2 tumors for MMP-1 receptor. Immunofluorescence images of cell nucleus blue (first column), mouse MMP-1 red (second column), and merged (third column) MMP-1 and cell nucleus-stained image (the overlap of blue and red fluorescence confirms the co-localization of MMP-1) proved the expression of MMP-1 on plasma membrane in HEp-2 cells. MMP-1 was visualized with Cy3 dye (red). Cell nuclei were stained with DAPI (St. Louis, MO, USA) (blue). Scale bar = 200 µm.

**Figure 7:**
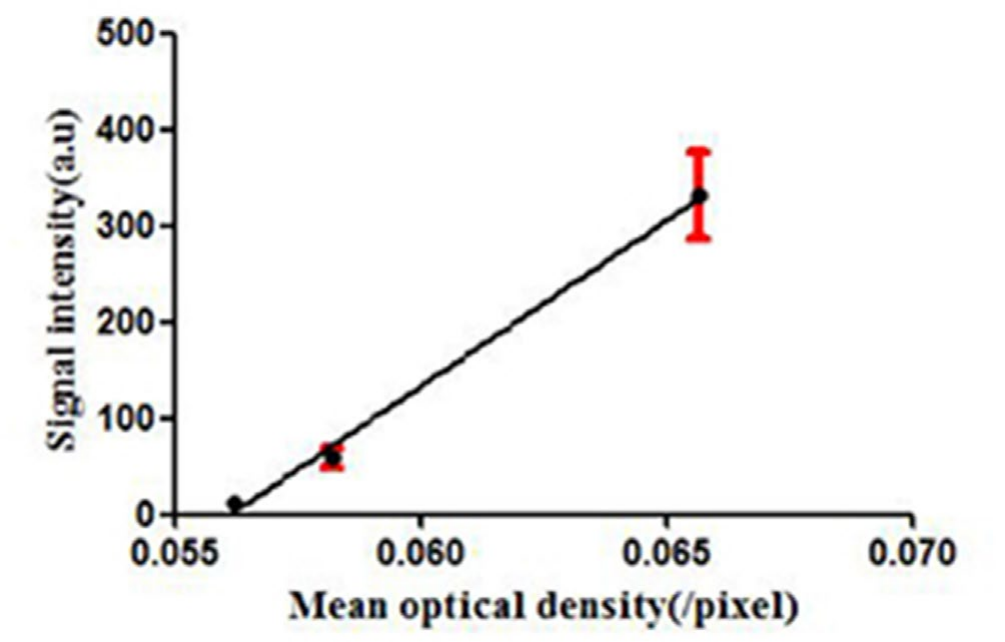
dTE signal intensity of USMI and mean optical intensity (/pixel) images of tumor immunofluorescence staining separately at Day 7, Day 12, and Day 17.

To confirm the results of USMI using the MB_MMP-1_, the tumor slices at Day 7, Day 12, and Day 17 were executed and subsequently analyzed for MMP-1 expression by immunofluorescence. Immunofluorescence results are shown in **Figure 6**; we could clearly see that the MMP-1 (red) was highly expressed in LSCC, confirming the presence of mouse MMP-1 on plasma membrane within LSCC in our study. In addition, we did the correlation analysis between the dTE signal intensity of USMI and mean optical density (/pixel) of MMP-1 expression in **Figure 7**; it was obvious that the dTE signal intensity of USMI was linearly enhanced as the mean optical density under the growth of the tumor. On the whole, the results of immunofluorescent evaluation at Day 7, Day 12, and Day 17 are relevant to the dTE signal intensity acquired with the MB_MMP-1_ by USMI.

## Discussion and Conclusion

Previous research had developed and tested arginine-glycine-aspartate (RGD)-MBs using αvβ3 integrin for a biomarker of the neovasculature in HEp-2 mouse tumor model (Hu et al., 2016). It had been proven that αvβ3 integrin-targeted RGD-MBs could effectively assess the expression of neovasculature and enhance the contrast imaging signal. However, the specificity of αvβ3-targeted MBs for LSCC detection was still not high enough because of its universality of expression in various types of malignant tumors (Sarmishtha et al., 2003; Brown et al., 2004; Hall and Jeffrey, 2004). Despite the growing number of published studies on USMI, a main obstacle for a clinical early diagnosis still exists.

In 1999, Maniotis and colleagues found the existence of VM in human melanoma cells (Maniotis et al., 1999). Subsequently, more and more studies had confirmed that VM is the new generation of tumor microcirculation without the participation of endothelial cells (Maniotis et al., 1999; Wang et al., 2010). And in tumor environment, it also was of importance to LSCC aggression and facilitates distant metastasis (Maniotis et al., 1999; Quail and Joyce, 2013). On the basis of these events, we assume that the lipid-shelled MBs loaded with mouse anti-MMP-1 monoclonal antibodies could tightly gather around VM to evaluate the expression of MMP-1 in LSCC.

In our current study, a novel MB_MMP-1_ USMI agent with C_3_F_8_-filled lipid-shelled MBs were prepared to dynamically evaluate the expression of MMP-1 in LSCC separately at Day 7, Day 12, and Day 17. First, we found that the MB_MMP-1_ had the ideal particle size range and that 1 × 10^8^ MBs/mL concentration enhanced the US imaging signal *in vitro*. Then, we further confirmed the specific binding ability of MB_MMP-1_ to HEp-2. It revealed that MB_MMP-1_ not only exhibited significantly greater adhesion to HEp-2 than did control MB_IgG_ but also significantly provided a prospecting result for the next animal experiment. *In vivo*, we clearly found that the signal intensity of dTE of MB_MMP-1_ group had significantly higher retention than did control MB_IgG_ between each group. In addition, it is also noticeable that the signal intensity of dTE showed an upward trend with the time increasing of tumor. This result could be because a much more universal VM was newborn in the later period of LSCC than in the early period (Wang et al., 2010). A further study also confirmed that more and more newborn VM were increasing the participates in cancer progression and metastasis to supply tumor with sufficient nutrition (Hong and Hui, 2018). As expected, these *in vivo* dynamical evaluation results are further confirmed by immunofluorescence. From **Figure 6**, the red-stained MMP-1 receptors were much more expressed with the growth of tumors. This immunofluorescence analysis was well correlated with the results from signal intensity of dTE by USMI *in vivo* (**Figure 7**).

The following limitations of the study need to be solved. First, the size of MBs we chose is of microsize level, which can only indirectly draw support from no endothelial cells’ VM by USMI. Second, although there was a strong significance in evaluating the expression of MMP-1 in LSCC, contrast agents adhering to more than one molecular biomarker may be advantageous over single-targeted contrast agents (Du et al., 2018) by increasing the number of MBs attached at sites of tumor neovascular and tumor tissue.

In conclusion, a novel MB_MMP-1_ US contrast agent will lay the foundation for the application of target biomarker of LSCC for USMI and will be a promising method to improve the early diagnosis.

## Ethics Statement

All animal studies were approved by the Institute’s Animal Care and Use Committee of Shenzhen Second People’s Hospital, Guangzhou University School of Medicine, China.

## Author Contributions

QH and ZL proposed the project. YZ and ZS conducted the study, XJ, HZ, and XW analyzed the data. YZ wrote the manuscript. QH revised the manuscript. All authors reached an agreement with the final version of the manuscript.

## Funding

The present study received financial support from The National Natural Science Foundation of China (81660292) and Shenzhen Science and Technology Project (project numbers JCYJ20170817171836611 and JCYJ20170306092258717). The National Natural Science Foundation of China (81260223 and 81301300) and GuangXi medical high-level backbone personnel training “139” project also supported this study.

## Conflict of Interest Statement

The authors declare that the research was conducted in the absence of any commercial or financial relationships that could be construed as a potential conflict of interest.
